# Gene regulation network analysis reveals core genes associated with survival in glioblastoma multiforme

**DOI:** 10.1111/jcmm.15615

**Published:** 2020-07-21

**Authors:** Lan Jiang, Min Zhong, Tianbing Chen, Xiaolong Zhu, Hui Yang, Kun Lv

**Affiliations:** ^1^ Central Laboratory Yijishan Hospital of Wannan Medical College Wuhu China; ^2^ Key Laboratory of Non‐coding RNA Transformation Research of Anhui Higher Education Institution Wannan Medical College Wuhu China

**Keywords:** CCNB2, CDC20, glioblastoma, MYBL2, RRA

## Abstract

Glioblastoma multiforme (GBM) is a very serious mortality of central nervous system cancer. The microarray data from GSE2223, GSE4058, GSE4290, GSE13276, GSE68848 and GSE70231 (389 GBM tumour and 67 normal tissues) and the RNA‐seq data from TCGA‐GBM dataset (169 GBM and five normal samples) were chosen to find differentially expressed genes (DEGs). RRA (Robust rank aggregation) method was used to integrate seven datasets and calculate 133 DEGs (82 up‐regulated and 51 down‐regulated genes). Subsequently, through the PPI (protein‐protein interaction) network and MCODE/ cytoHubba methods, we finally filtered out ten hub genes, including FOXM1, CDK4, TOP2A, RRM2, MYBL2, MCM2, CDC20, CCNB2, MYC and EZH2, from the whole network. Functional enrichment analyses of DEGs were conducted to show that these hub genes were enriched in various cancer‐related functions and pathways significantly. We also selected CCNB2, CDC20 and MYBL2 as core biomarkers, and further validated them in CGGA, HPA and CCLE database, suggesting that these three core hub genes may be involved in the origin of GBM. All these potential biomarkers for GBM might be helpful for illustrating the important role of molecular mechanisms of tumorigenesis in the diagnosis, prognosis and targeted therapy of GBM cancer.

## 1 INTRODUCTION

Glioblastoma multiforme (GBM) is an incurable malignancy.^1^ Almost all GBMs recur within the first year following diagnosis, the recurrence rate of GBM is particularly high, they can be surgically resected again.^2^ Heterogeneity is the key point for the treatment of glioma.^3^ It is still very difficult to deal with the recurrence of GBM during the treatment. Based on the histology, gliomas would be classified into WHO (World Health Organization) grades I, II, III and IV.^4^ The natural course of low‐grade glioma (WHO Grade II) is to transform or to dedifferentiate into high‐grade glioma (WHO grade III–IV), and they recur after surgical resection frequently. The limited information on the pathogenesis, development, reproduction and molecular mechanisms of GBM has hindered the research and development of precise treatment of available drugs. Therefore, it is urgent to clarify the relevant molecular mechanisms of GBM and actively develop new therapeutic strategies.

Many studies have illustrated the numerous candidate hub genes involved in GBM from RNA‐seq data. Biomarkers could combine with different diseases and display a high value which lead to the development of a robust, effective take on GBM therapy.^5^ Numerous recent studies have discovered the biomarkers in GBM from molecular biology to proteomics, such as circulating tumour DNA (ctDNA), DNA, microRNA (miRNA), lncRNA (long non‐coding RNA) and protein.^6^, ^7^, ^8^ ctDNAs in cerebrospinal fluid better reflected the sequential change of those tumour drivers than in plasma, which served as biomarkers to improve patients’ outcomes.^9^ GFAP (Glial Fibrillary Acidic Protein) and EGFR (Epidermal Growth Factor Receptor) were considered to be increased and as potential therapeutic markers in GBM patients.^10^, ^11^ CDK1 (Cyclin Dependent Kinase 1) and BUB1 (BUB1 Mitotic Checkpoint Serine/Threonine Kinase) were significantly connected with carcinogenesis of GBM.^12^, ^13^ Hsa‐miR‐21 and hsa‐miR‐10b were discovered as GBM‐specific miRNAs, which lead to the development of a robust take on GBM therapy.^5^ The blood biomarkers (LRG1 (Leucine Rich Alpha‐2‐Glycoprotein 1), CRP (C‐Reactive Protein) and C9 (Complement C9)) revealed significant positive correlations with tumour size.^14^ Our previous study also reported that HMG‐box family and related ceRNA (competing endogenous RNA) established the significance of SOX6 (SRY‐Box Transcription Factor 6) in the malignant progression of glioblastoma.^6^

Taking into account the individual differences, in this study, we selected the microarray data from the Gene Expression Omnibus (GEO) database and the RNA‐seq data from TCGA‐GBM (The Cancer Genome Atlas Glioblastoma Multiforme) dataset, using the RRA (RobustRankAggreg) method to identify differentially expressed genes (DEGs) between GBM tissues and normal tissues. Through network analysis and functional analyses, it is possible to predict the pathways and interactions of DEGs. In addition, hub genes related lncRNA, miRNA and transcription factor (TF) were also explored. All of these bioinformatics methods were used to elucidate the comprehensive molecular mechanisms responsible for the development and progression of GBM and to provide potential biomarkers as a treatment for patients in different subgroups of GBM.

## 2 METHODS AND MATERIALS

### 2.1 Data preparation and RRA method analyses

The microarray data of human tissue from GSE2223,^15^ GSE4058,^16^ GSE4290,^17^ GSE13276,^18^ GSE68848 ^19^ and GSE70231 ^20^ were downloaded from the Gene Expression Omnibus (GEO) database (https://www.ncbi.nlm.nih.gov/geo/). (a) The inclusion and exclusion criteria were applied for the selection of GEO datasets (inclusion: datasets containing tumour and normal samples; exclusion: experiments on cell lines, datasets containing serum samples nor tissue biopsies, etc). (b) They were GBM and normal tumours cases based on 456 samples connected with subtypes (389 tumours and 67 normal samples). GSE2223 (29 GBM samples: four normal samples, GPL1833); GSE4058 (30 GBM samples: 3 normal samples, GPL182); GSE4290 (76 GBM samples: 23 normal samples, GPL570); GSE13276 (5 GBM samples: 3 normal samples, GPL96); GSE68848 (228 GBM samples: 28 normal samples, GPL570); GSE70231 (21 GBM samples: 6 normal samples, GPL80). (c) Limma package,^21^ Deseq2^22^ and edgeR method^23^ (fold change > 2 and adjusted *P*‐value (*q*‐value) < .05) were used for identifying GEO data DEGs (differential expressed genes), absolute value of fold change > 2 and adjusted *P*‐value (*q*‐value) < .05 were considered as DEGs. The DEGs result of each dataset was drawn the violin plot by using ggplot2 package,^24^ respectively.

Then, we downloaded the RNA‐seq data of human tissue from TCGA‐GBM dataset (169 GBM and five normal samples)^25^ and identified DEGs by intersecting with the results with limma, Deseq2^22^ and edgeR method^23^ (fold change > 2 and *q*‐value < .05).

Finally, RobustRankAggreg package (RRA method) was designed to sort the multi‐gene lists and adopted to gain the robust DEGs.^26^ The pheatmap package is used to visualize the top 20 up‐ and top 20 down‐ DEGs obtained by the six GEO datasets and TCGA‐GBM dataset by RRA method.^27^, ^28^

### 2.2 Function and network analyses

Protein‐protein interaction (PPI) network was obtained from STRING v11.^29^ We used the Cytoscape v 3.7.2 plugin MCODE and cytoHubba to select the hub genes.^30^ We used Cytoscape to visualize the PPI networks, and MCODE plugin to screen a significant module from the PPI network with a degree cutoff = 2, node score cutoff = 0.2, node density cutoff = 0.1, Max depth = 100 and K‐core = 2. Then, cytoHubba plugin was used to determine the hub genes when the degrees were ≥ 10.

The analyses of GO (Gene Ontology) enrichment, KEGG (Kyoto Encyclopedia of Genes and Genomes) pathway were performed via clusterProfiler package.^31^ Gene set enrichment analysis (GSEA) was conducted by clusterProfiler package as well and drawn as emapplot and heatplot to better understand the results for GSEA. Survival analysis was drawn based on GlioVis database (http://www.gliovis.bioinfo.cnio.es). Tumour‐infiltrating immune cells were inferred using TIMER (Tumor Immune Estimation Resource).^32^ Functional associations of the hub genes (with TF, miRNAs and lncRNAs) were analysed using NetworkAnalyst.^33^

### 2.3 Validation of core hub genes

We downloaded the RNA‐seq data of human tissues from CGGA, after batching all mRNA‐seq matrix and removing the incomplete and duplicate data, we analysed these data by R packages: (a) Prognostic accuracy of the three core hub genes evaluated by ROC (receiver operating characteristics) curve with respect to 1 year, 3 year and 5 year survival of glioma patients by ‘survivalROC’ package ^34^; (b) Drawing forest plots of univariate and multivariate cox analysis by ‘survival’ package ^35^; (c) The expression of core hub genes in different types by ‘beeswarm’ package.^36^ Next, we validated core hub gene in HPA (The Human Protein Atlas) (GBM U251‐MG cell line) (https://www.proteinatlas.org) and CCLE (Cancer Cell Line Encyclopedia) (glioma cell) database (https://portals.broadinstitute.org/ccle).

## 3 RESULTS

### 3.1 The overall collection of datasets and identification of DEGs

**Figure 1:**
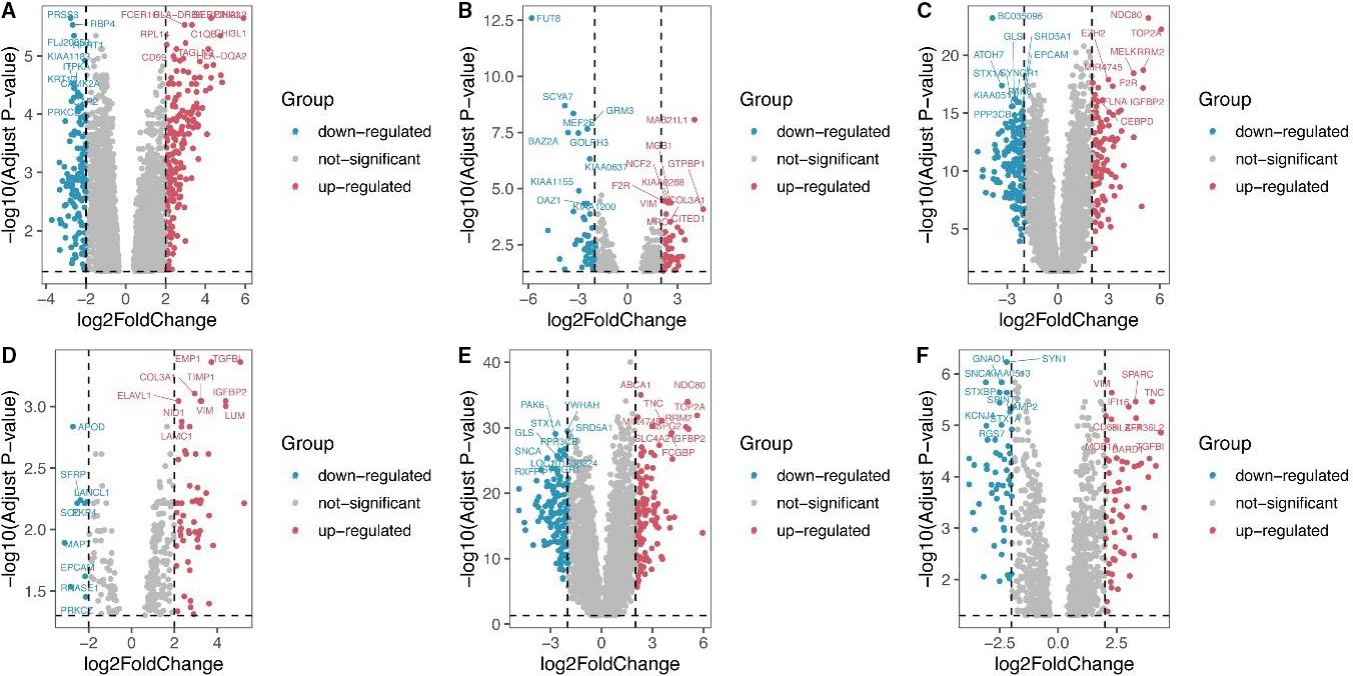
Volcano plots for DEGs in GBM and normal tissues based on data from the GEO datasets. A. GSE2223, B. GSE4058, C. GSE4290, D. GSE13276, E. GSE68848 and F. GSE70231

Six gene datasets were collected from NCBI (National Center for Biotechnology Information) GEO database (GSE2223, GSE4058, GSE4290, GSE13276, GSE68848 and GSE70231) and TCGA‐GBM dataset (Table 1). A total of 389 GBM tumour and 67 normal tissues were adopted in this study. With t

he criteria of log2 (fold change)> 1 and adjusted *P*‐value < 0.05, 754 up‐regulated and 750 down‐regulated DEGs were conducted in GSE2223 (Figure 1A); 176 up‐regulated and 174 down‐regulated DEGs were gained in GSE4058 (Figure 1B); 566 up‐regulated and 487 down‐regulated DEGs were obtained in GSE4290 (Figure 1C); 177 up‐regulated and 51 down‐regulated DEGs were gained in GSE13276 (Figure 1D); and 619 up‐regulated and 492 down‐regulated DEGs were adopted in GSE68848 (Figure 1E); and 377 up‐regulated and 244 down‐regulated DEGs were obtained in GSE70231 (Figure 1F). In TCGA‐GBM dataset, we used three methods to find DEGs, there were 2335 DEGs in edgeR, 1303 DEGs in limma, and 1555 in Deseq2, then we took the intersection of the three methods, and 1255 DEGs were obtained from TCGA‐GBM in final.

**Table 1:** The selected samples for this current study

GEO accessions	GBM number	Normal number	Reference
GSE2223	29	4	15
GSE4058	29	3	16
GSE4290	77	23	17
GSE13276	5	3	18
GSE68848	228	28	19
GSE70231	21	6	20
TCGA‐GBM	169	5	25

**Figure 2:**
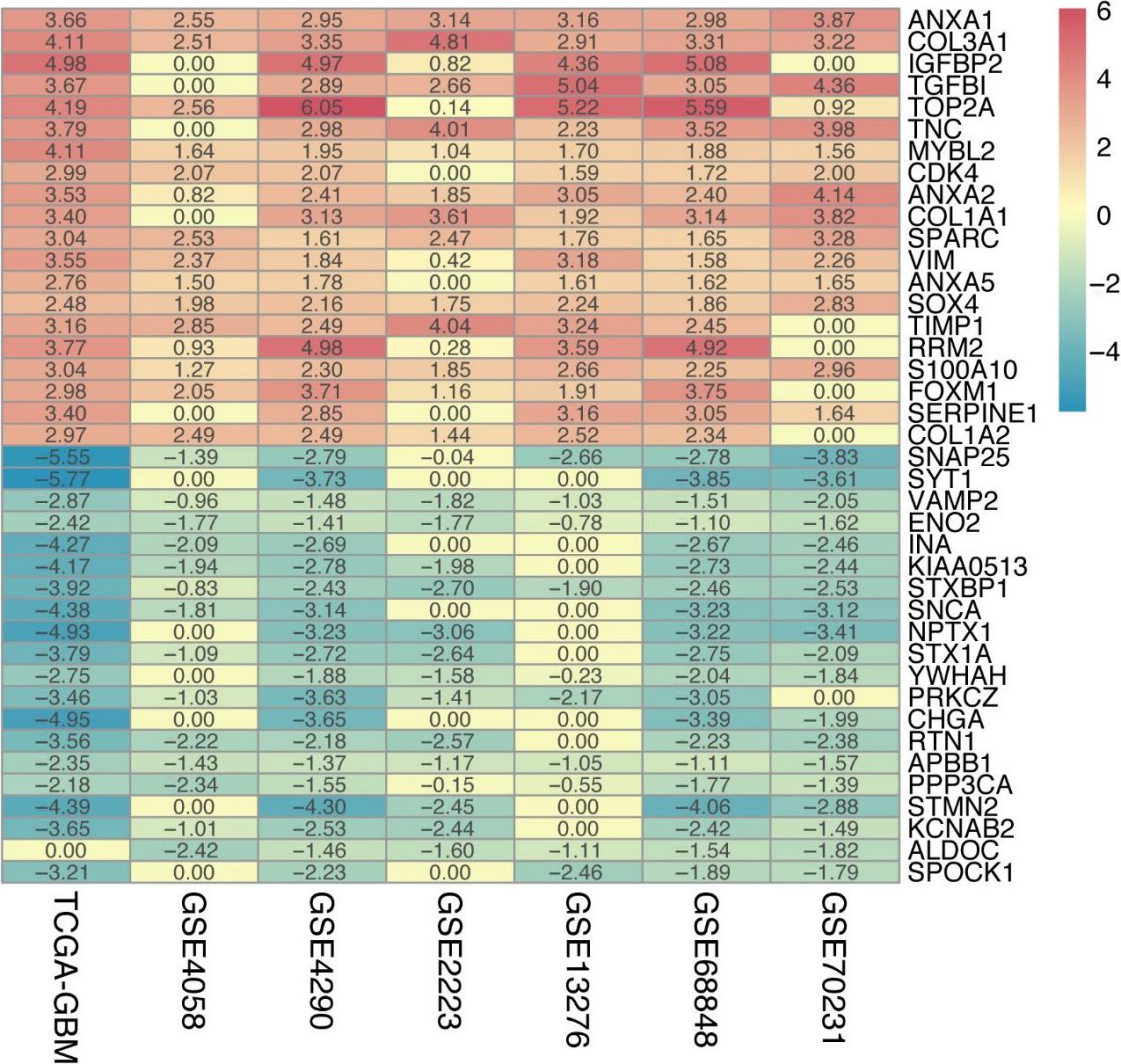
Heat map for the 20 top up‐regulated and 20 top down‐regulated DEGs in GBM using the RobustRankAggreg method with *q*‐value < .05 and fold change > 2

The RRA method was used to identify genes that are ranked consistently better and to explore the robust DEGs in different seven datasets. Finally, we determined 133 significantly DEGs in these datasets, including 82 up‐regulated and 51 down‐regulated DEGs (Table S1). The expression heat map of the top 20 up‐regulated and top 20 down‐regulated DEGs is visualized in Figure 2.

### 3.2 PPI network and hub gene detection

**Figure 3:**
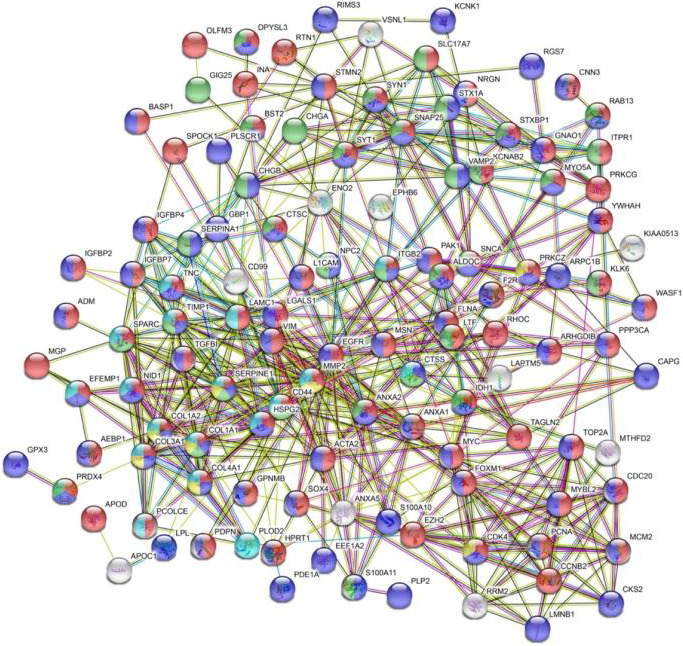
PPI network of the DEGs in GBM. The top one biological processes (BP), molecular functions (MF), cellular components (CC), KEGG pathway and Reactome pathway were marked as red, purple, green, yellow and blue, respectively

These 133 DEGs were input to the STRING database for PPI networks, for their potential biological functions. The clusters of sub‐networks that were obtained from STRING database with 133 nodes and 547 edges (PPI enrichment *P*‐value < 1.0e‐16) (Figure 3).

The top one biological processes (BP), molecular functions (MF) and cellular components (CC) were listed in Table S2 and marked as red, purple and green, respectively. The highest enriched BP, MF, CC terms were ‘developmental process’, ‘protein binding’, ‘secretory vesicle’. The top one KEGG pathway was ‘AGE‐RAGE signalling pathway in diabetic complications’ marked as yellow. In the glioma pathway (hsa05214), included in CDK4 (Cyclin Dependent Kinase 4), PRKCG (Protein Kinase C Gamma) and EGFR. The highest enriched Reactome pathway was ‘Extracellular matrix organization’ marked as blue (Figure 3 and Table S2).

**Figure 4:**
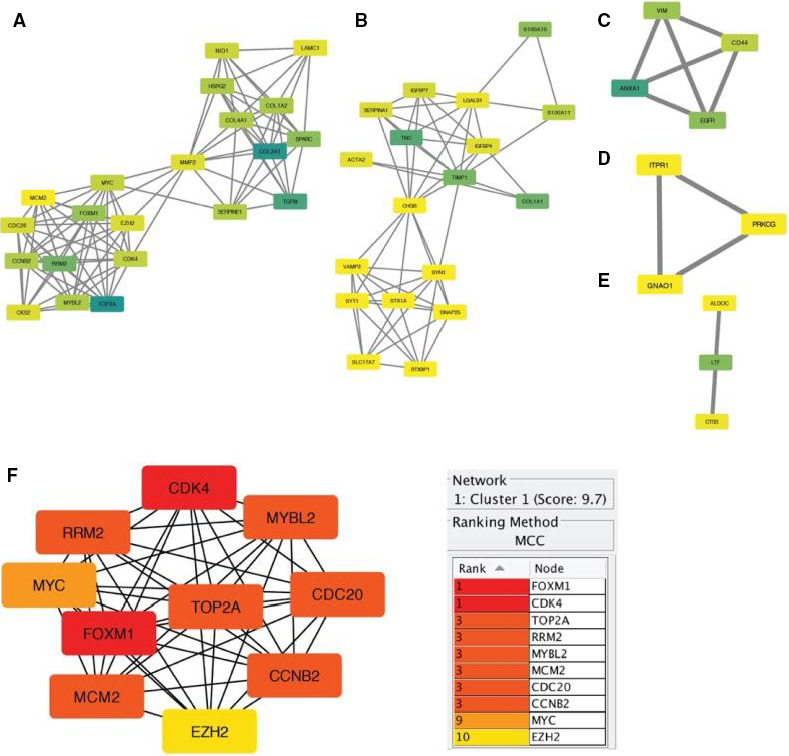
Hub genes screening from MCODE and cytoHubba plugin in cytoscape v3.7.2. A‐E, MCODE results; F, cytoHubba result

Next, Cytoscape plugin MCODE were used to find out the hub genes. We found five types from MCODE results, the cluster one with score = 9.7, obtained 21 DEGs, and the other four clusters with score = 6.588, 4, 3 and 3, respectively (Figure 4A‐E). Through MCODE analysis results, we used cytoHubba for further analysis in the results of cluster one (21 DEGs), we determined the top 10 DEGs as hub genes (FOXM1 (Forkhead Box M1), CDK4, TOP2A (DNA Topoisomerase II Alpha), RRM2 (Ribonucleotide Reductase Regulatory Subunit M2), MYBL2 (MYB Proto‐Oncogene Like 2), MCM2 (Minichromosome), CDC20 (Cell Division Cycle 20), CCNB2 (Cyclin B2), MYC (MYC Proto‐Oncogene, BHLH Transcription Factor) and EZH2 (Enhancer of Zeste 2 Polycomb Repressive Complex 2 Subunit)) (Figure 4F).

### 3.3 Functional and survival analyses

**Figure 5:**
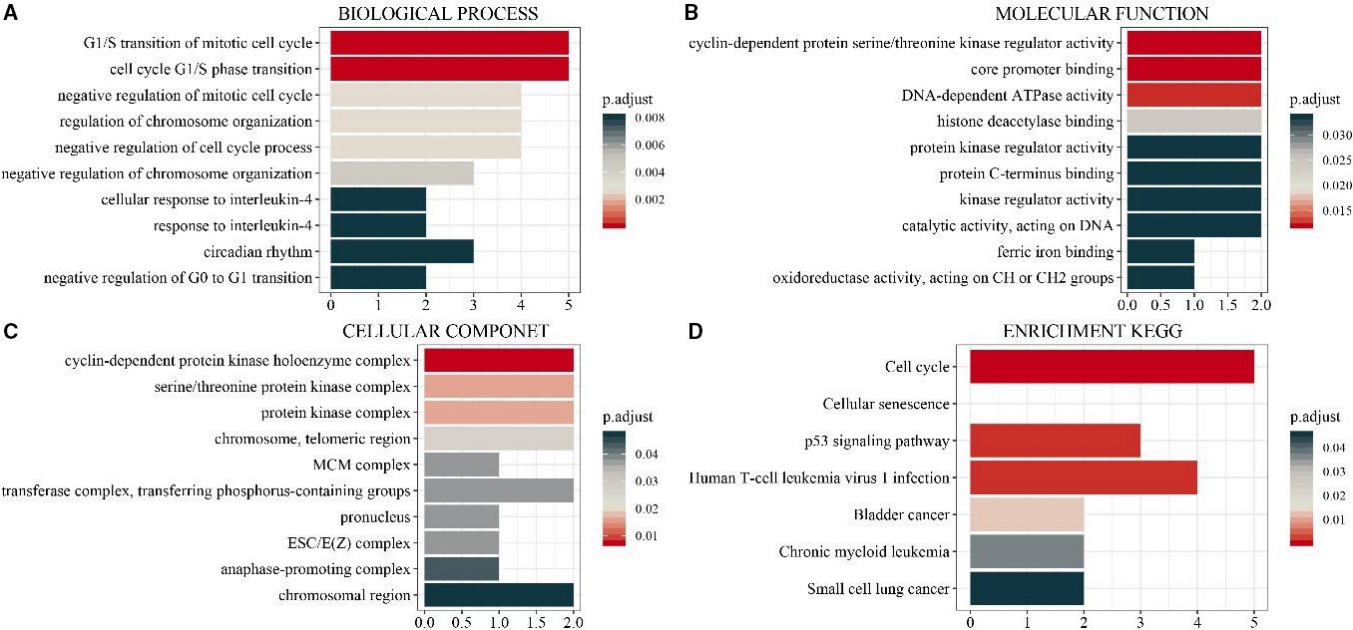
GO enrichment annotations and KEGG pathways of the DEGs in GBM. A, Top 10 biological process terms. B, Top 10 molecular function terms. C, Cellular component terms. D, significantly enriched KEGG pathways (adjusted *P*‐value < .05)

**Figure 6:**
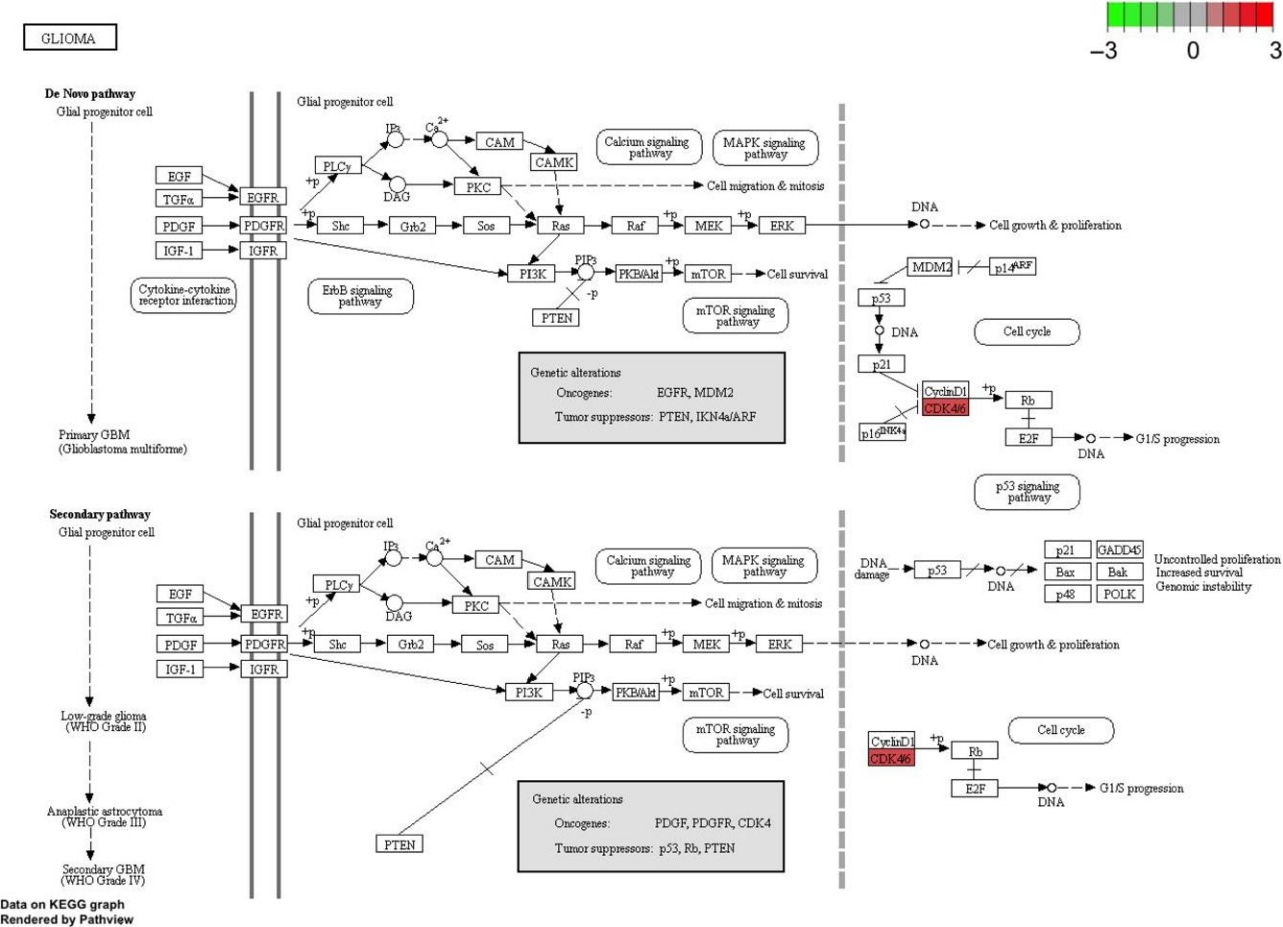
Glioma pathway from KEGG analysis which was performed by pathview package. One hub gene CDK4 in glioma pathway marked as red colour

Gene ontology (GO) functional enrichment analyses were used to determine the potential molecular mechanisms employed by these 10 hub genes. The top 10 biological processes (BP), molecular functions (MF) and cellular components (CC) are listed in Figure 5A‑C. The highly enriched BP terms were ‘G1/S transition of mitotic cell cycle’, ‘cell cycle G1/S phase transition’ and ‘negative regulation of mitotic cell cycle’. The markedly enriched MF terms were ‘cyclin‐dependent protein serine/threonine kinase regulator activity’, ‘core promoter binding’ and ‘DNA‐dependent ATPase activity’. The predominantly enriched CC terms were ‘cyclin‐dependent protein kinase holoenzyme complex’, ‘serine/threonine protein kinase complex’. With adjusted *P*‐value < 0.05, seven pathways were enriched by the 10 DEGs (Figure 5D), many of which were tumour‑associated pathways, including the ‘Bladder cancer’, and the ‘p53 signalling pathway’ and the ‘Small cell lung cancer’. Moreover, CDK4 was predicted to be centralized in the glioma pathway (hsa05214) (Figure 6).

**Figure 7:**
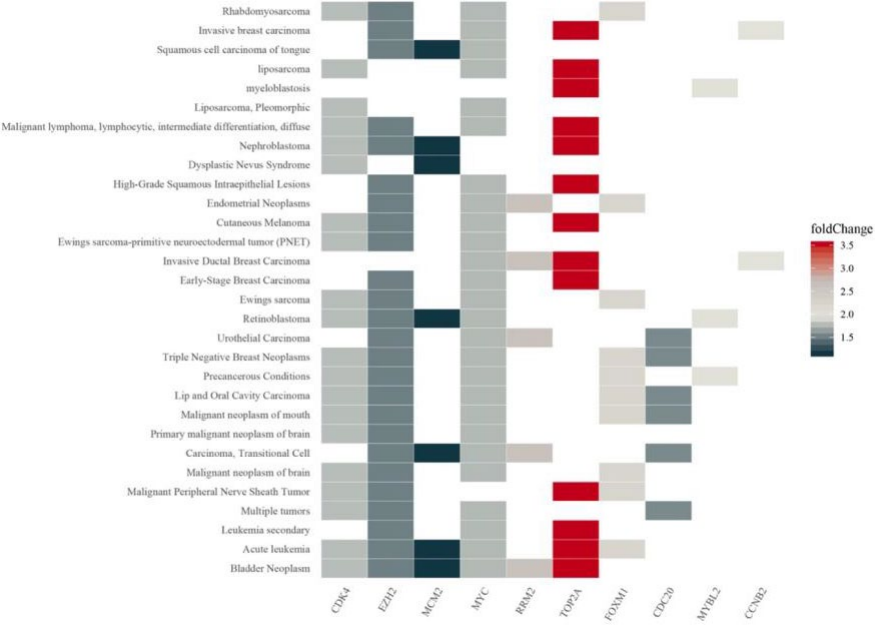
GSEA heatplot of the hub genes expression profiles using clusterProfiler

GSEA heatplot of enriched DEGs list on each term was shown in Figure 7 and Figure S2. There are four types (primary malignant neoplasm of brain, malignant peripheral nerve sheath tumour, malignant neoplasm of brain, ewings sarcoma‐primitive neuroectodermal tumour (PNET)) related to nervous system diseases. In primary malignant neoplasm of brain which including three DEGs (CDK4, EZH2, MYC). We could also find 4 DEGs (CDK4, EZH2, FOXM1 and TOP2A) in the type of malignant peripheral nerve sheath tumour. In addition, CDK4 and EZH2 were determined to be centralized in all four types (Figure 7).

**Figure 8:**
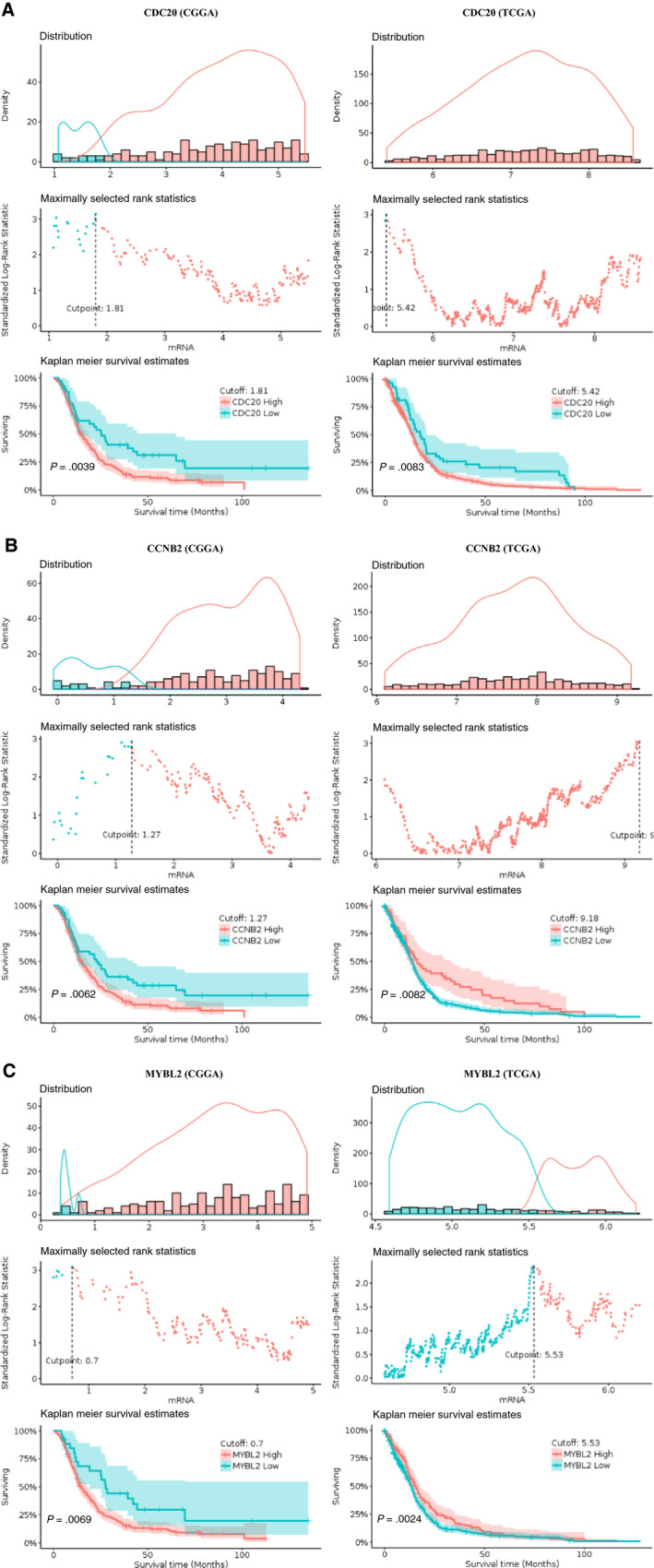
Kapla‐Meier plots for DEGs to visualize the survival differences using GlioVis database

As shown in Figure 8, based on GlioVis database (TCGA‐GBM and CGGA‐GBM), CDC20, CCNB2 and MYBL2 (*P*‐value < 0.01) (Figure 8) was correlated with the survival of determine optimal cutoff for Kaplan‐Meier survival analysis (Figure 8). GlioVis used the maximally selected statistics to determine the optimal cutoff for continuous variables, which provided in the ‘survminer’ package. CDC20, CCNB2 and MYBL2 were highly expressed in GBM samples. Thus, above three predicted hub genes might play important roles in ‘Cellular senescence’ and ‘Cell cycle’ and were considered potential prognostic biomarkers in GBM and were the subject of further study.

### 3.4 Tumour‐infiltrating immune cells analyses and target interactive analyses

We used hub genes to predict immune‐cell profiling in GBM by TIMER, a web tool to analysze infiltrated immune cells in the TCGA dataset. And we conducted the abundance of six types of tumour‐infiltrating immune cells (B cells, CD4 + T cells, CD8 + T cells, neutrophils, macrophages and dendritic cells) and purity (Figure S3). The expression levels of RRM2, MYC and MCM2 were significantly correlated with dendritic cell (DC) infiltration. We detected that MCM2 had a positive correlation with the purity of GBM and was highly correlated to DCs. The corrections among the 10 hub genes in GBM were shown in Figure S4.

**Figure 9:**
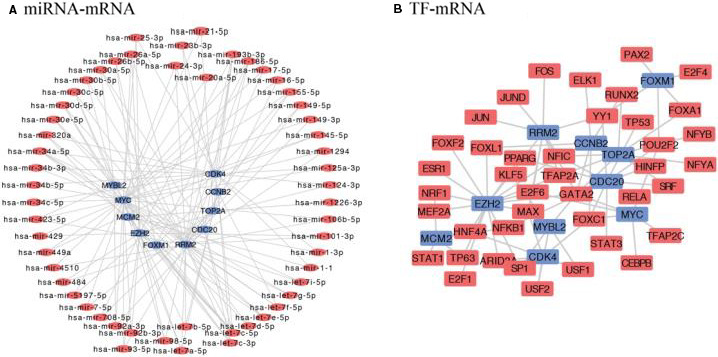
TF and miRNA with seven hub gene and their regulatory network. Blue shows hub genes, circle red shows miRNAs, square red means TF

Subsequently, we predicted the target of ten hub genes and predicted their network interaction with lncRNA, miRNA and transcription factors (TF) (Figure 9 and Table S3). A total of 88 lncRNAs could target the ten hub genes (Table S3), such as HNF1A‐AS1 could target EZH2 and MYC. In Figure 9A, CDK4 was targeted by hsa‐miR‐21‐5p and hsa‐miR‐193b‐3p. We also found TOP2A could regulate RRM2, TOP2A, CDC20 and EZH2 expression (Figure 9B). FOXM1 could interact with PAX2 (Paired Box 2) and E2F4 (E2F Transcription Factor 4). Combined the results of survival analysis and lncRNA‐, miRNA‐ and TF network analysis, we were interested to further illustrate the molecular mechanism that were regulated these ten hub genes.

### 3.5 Validation in the CGGA, HPA and CCLE database: CDC20, CCNB2, MYBL2

**Figure 10:**
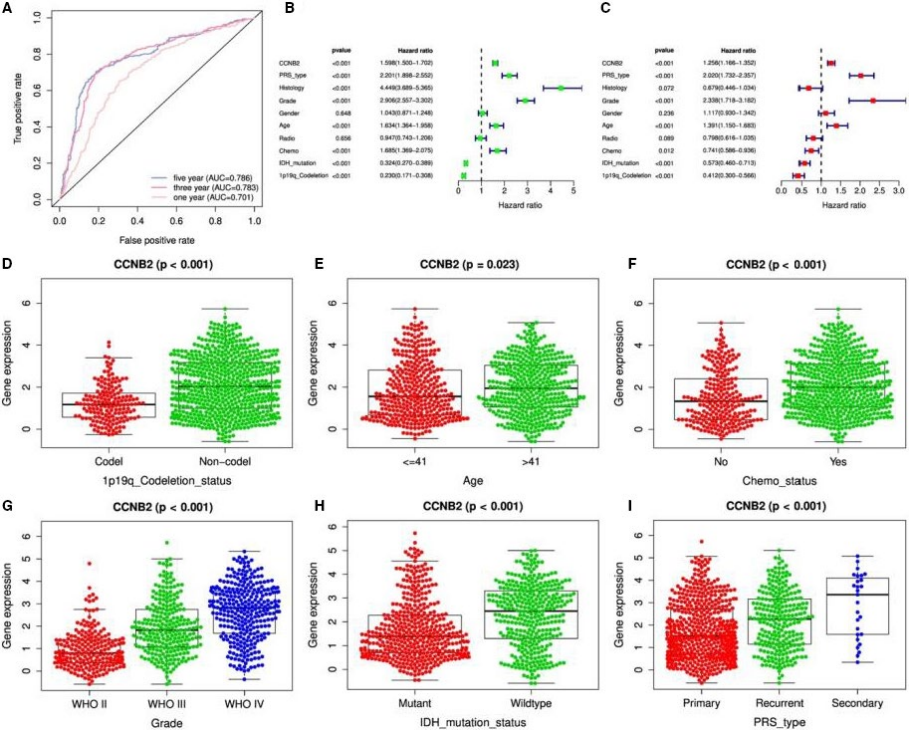
CCNB2 validated in CGGA database. A, Prognostic accuracy of the CCNB2 evaluated by the AUC of the time‐dependent ROC with respect to 1, 3 and 5 year survival of glioma patients in the CGGA dataset; B and C, Univariate and multivariate cox analysis of CCNB2 in CGGA; D. to J. The expression of CCNB2 in different types

We further validated these ten central genes in the CGGA database and detected that CCNB2, CDC20 and MYBL2 expression was regular in different tissues (Figure 10, Figures S5 and S6). Subsequently, we evaluated the prognostic accuracy of CCNB2, CDC20 and MYBL2 by calculating the time‐dependent ROC, AUC (area under the ROC curve) for one‐, three‐ and five‐year survival in glioma patients. CCNB2, CDC20 and MYBL2 showed good prognostic accuracy for the CGGA dataset (Figure 10A, Figures S5A and S6A). After univariate and multivariate cox analysis of key clinical and molecular factors, we found that CCNB2 expression (*P*‐value < 0.001, HR = 1.598/1.256), and CDC20 expression (*P*‐value < 0.001, HR = 1.606/1.246), and MYBL2 expression (*P*‐value < 0.001, HR = 1.570/1.258) were independent prognostic factors for gliomas in the CGGA dataset (Figure 10B‐C, and Figures S5B,C and S6B,C). Because of the IDH1 mutation status and WHO classification are important for the prognosis of GBM, so it is necessary to determine whether our risk score is an independent prognostic factor for overall survival. The prognostic significance of IDH1 mutation status and 1p19q codeletion status in GBM in the CGGA database was shown to be highly significant. When GBM patients in the CGGA dataset were classified into two groups according to CDC20, CCNB2 and MYBL2 expression for survival analysis. For core hub genes, the expression levels of CCNB2, CDC20 and MYBL2 were also significantly higher in GBM with IDH1 mutations than in those of IDH1 wild‐type (Figure 10H and Figures S5H and S6H). CCNB2, CDC20 and MYBL2 can classify patients into high‐risk and low‐risk groups according to different 1p19q_codeletion states (Figure 10D and Figures S5D and S6D), age (Figure 10E and Figures S5E and S6E) and chemical status (Figure 10F and Figures S5F and S6F), WHO ranks (Figure 10G and Figures S5G and S6G), IDH1 states (Figure 10H and Figures S5H and S6H) and PRS (main, recurrent, second category) Type (Figure 10I and Figures S5I and S6I), which p‐value < 0.05. After univariate and multivariate cox analysis of core hub genes (CDC20, CCNB2 and MYBL2), we found these three genes independently indicated unfavourable prognosis in CGGA database, the expression of CCNB2, CDC20 and MYBL2 in different types could be independent prognostic marker in GBM.

**Figure 11:**
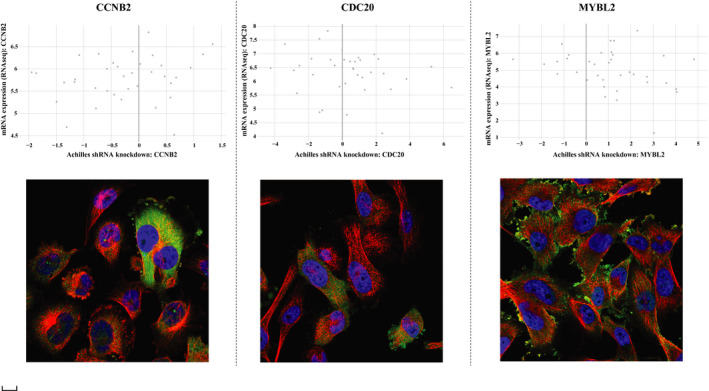
Validation of core hub genes in CCLE and HPA database. A, CCNB2; B, CDC20; C, MYBL2. The upper part of figure means the comparison of the RNA‐seq expression in the glioma cells and the Achilles shRNA knockdown gene. The X axis is achilles shRNA knockdown: core gene, Y axis means mRNA expression (RNA‐seq): core gene. The lower part of figure shows immunofluorescent staining of human cell line U251‐MG, green represents antibody, red means microtubules, blue means nucleus. The scale bar was 20 μm

We selected CCLE and HPA database to further validate the core hub genes. In glioma cells from CCLE, we could see the expression of core gene both in RNA‐seq expression and Achilles shRNA knockdown (Figure 11). In GBM U251‐MG cells from HPA, immunofluorescent staining of human cell line U251‐MG showed us the gene location, green represents antibody, red means microtubules. CDC20 detected in the nucleoplasm and cytosol (Figure 11A), compared with other RNA cell lines, although the gene expression is not cell‐dependent, the expression of CDC20 in U251‐MG is the highest (Figure S7). CCNB2 localized to the cytosol and the Golgi apparatus, cell cycle dependent gene expression according to correlation analysis (Figure 11B). The localization of MYBL2 is the nucleoplasm (Figure 11C).

## 4 DISCUSSION

Recent bioinformatic studies aimed to analyse differentially expressed genes, miRNAs and lncRNAs demonstrated the robustness of the results obtained through the integration of different GEO and TCGA datasets,^37^, ^38^ encouraged researchers to carry out the real‐time analysis of in‐motion big data, while protecting privacy and security.^39^ With the development of sequencing and bioinformatics technology, more and more gene datasets are released on the public platform, we need to sort them out, and analysed them from more angles.^27^, ^40^, ^41^ Compared with low‐grade disease, hsa‐miR‐506‐514 cluster, hsa‐miR‐592, hsa‐miR‐199a‐5p were related to the overall survival of the uveal melanoma patients.^42^ Up‐regulated hsa‐miR‐183‐5p and down‐regulated hsa‐miR‐195‐5p were directly related to colorectal cancer in the cancer development.^43^ Increased C‐MYC associated with glucocorticoid and was resistant in acute lymphoblastic leukaemia.^44^ In this study, we selected six GEO datasets (389 GBM tumour and 67 normal tissues) and TCGA‐GBM dataset (169 GBM and 5 normal samples), which integrated them by the RRA method, with the criteria of log2 (fold change) > 1 and adjusted p‐value < 0.05, we filtered 133 DEGs (82 up‐regulated and 51 down‐regulated) in totally. After PPI network and functional analysis, we identified ten DEGs (FOXM1, CDK4, TOP2A, RRM2, MYBL2, MCM2, CDC20, CCNB2, MYC and EZH2) as core genes in GBM. GO, KEGG and GSEA enrichment analyses were used to further explore pathways for the development and progression of GBM. TIMER immunity infiltration and survival analysis were validated in conjunction with the TCGA database. At the same time, we further verified the core genes in the CGGA, HPA and CCLE database.

FOXM1 (Forkhead Box M1) is a member of FOX family and located on the chr12p13.33, which emerged as a key molecule implicated in initiation and progression of cancer.^45^ FOXM1 is a high‐risk myeloma gene with poor prognosis, FOXM1 was up‐regulated between GBM and normal tissues, and enriched in the GO term: cell cycle arrest, G2/M transition of mitotic cell cycle, negative regulation of stress‐activated MAPK cascade, suggesting that FOXM1 might be a potential gene in gliomas development. MYBL2 (MYB Proto‐Oncogene Like 2) is a member of the MYB family of TF genes, is a key downstream factor of AKT/FOXM1 signalling to promote progression of human glioma.^46^ The expression of COL1A2 (Collagen Type I Alpha 2 Chain) in GBM could significantly improve survival benefit after aggressive treatment compared with the proneural patients.^47^ COL1A2 was associated with poor outcomes in GBM and validated to be significantly linked to poor prognosis in both TCGA and CGGA database,^48^ as well as our study, so the over expression of COL1A2 might be important to the development of GBM. CDC20 (Cell Division Cycle 20) was reported as a target for overcoming TMZ‐resistance (Temozolomide resistance) in GBM.^49^ CDC20 detected in the nucleoplasm and cytosol, compared with other RNA cell lines, although the gene expression is not cell‐dependent, the expression of CDC20 in U251‐MG GBM cell line is the highest. We also found CDC20 is overexpressed in GBM with a poor prognosis in GBM patients.

DC are the antigen‐presenting cells, pathways of DCs are important to control the development of immune response and decisions on vaccination.^50^ MYC (MYC Proto‐Oncogene, BHLH Transcription Factor) in GBM was highly correlated with DC (partial cor = 0.27, *P*‐value = 1.95e‐08), could control the immune response. Activation of the MYC signalling pathway in normal astrocytes exposed to GBM‐EV may be the mechanism by which GBM acquires a phenotype that promotes tumour progression,^51^ MYC was enriched in developmental process and protein binding in this study, was consistent with the predictions of this study.

The miRNAs found altered in GBM have been widely reported, hsa‐miR‐21 was overexpressed in the development and progression of GBM.^52^ Our previous study has established ceRNA network and identified miRNA, lncRNA and TF were glioma‐related molecules in GBM.^6^ In current study, hsa‐miR‐21‐5p was involved in the regulation of CDK4 and MYC, which known to be detected in the GBM development. Six lncRNA‐related ceRNA combined with four small molecule compounds were considered to help identify the regulatory functions of lncRNAs in the pathogenesis of GBM.^53^ Via recruiting EZH2, LncRNA HOTAIR modulated the chromatin architecture.^54^ We also found that lncRNA APTR and lncRNA H19 were up‐regulated in GBM which interacted with EZH2, played a role in inhibiting the cell proliferation and promoting the tumorigenesis. LncRNA HOTAIR could also down‐regulate EZH2 promoting cell cycle, cell proliferation and cell invasion. E2F4 was increased in GBM, which stimulated the GBM growth.^55^ FOXM1 could also interact with E2F4 in this study. It was confirmed that the miRNAs here identified are able to target the hub genes here identified (specially CDK and cycline families).

Ten hub genes were all overexpressed in GBM, functional enrichment results focused on these up‐regulated genes, there were many significant enrichment results. CDK4, MCM2 and MYC were response to G1/S transition of mitotic cell cycle (GO:0 000 082) in biological process and enriched in the cell cycle (hsa04110) in KEGG pathway. CDK4, RRM2 and CCNB2 were related to p53 signalling pathway (hsa04115), which might activate the p53 signalling pathway via CDK4, RRM2 and CCNB2, might also have regulatory effects in glioma cells. EZH2 (Enhancer of Zeste 2 Polycomb Repressive Complex 2 Subunit) was up‐regulated in GBM, lncRNA HOTAIR, APTR and H19 could interact with EZH2, which promoted cell cycle, cell proliferation, cell invasion and tumorigenesis in GBM.

In our study, CCNB2, CDC20 and MYBL2 were up‐regulated in GBM compared with control brain tissues, with poor prognosis in GBM patients. We found that mRNA expression of CCNB2, CDC20 and MYBL2 was significantly different in primary, recurrent and secondary GBM (primary > recurrent>secondary), suggesting that CCNB2, CDC20 and MYBL2 might be involved in the origin of GBM. The results described above indicated that CCNB2, CDC20 or MYBL2 was independent prognostic marker for overall survival and might play a significant role in determining glioma prognosis, and the association between CCNB2, CDC20, MYBL2 and GBM should be investigated further.

## 5 CONCLUSIONS

In summary, bioinformatics analysis in the context of big data identified key roles for ten hub genes FOXM1, CDK4, TOP2A, RRM2, MYBL2, MCM2, CDC20, CCNB2, MYC and EZH2 in the development, progression, diagnosis, treatment and prognosis of GBM. These results provide candidate gene for molecular targeting therapy and biomarker for radiotherapy of GBM cancer, and further in vivo and in vitro experiments are needed to validate the role of these screened genes and pathways. At the same time, further research is needed for the synergistic interaction between CDC20, CCNB2 and MYBL2.

## CONFLICTS OF INTEREST

The authors confirm that there are no conflicts of interest.

## AUTHOR CONTRIBUTION

**Lan Jiang:** Data curation (equal); Formal analysis (equal); Validation (equal); Writing‐original draft (equal); Writing‐review & editing (equal). **Min Zhong:** Formal analysis. **Tianbing Chen:** Formal analysis (equal); Funding acquisition (equal). **Xiaolong Zhu:** Formal analysis (equal). **Hui Yang:** Formal analysis (equal). **Kun Lv:** Data curation (equal); Funding acquisition; Writing‐review & editing (equal).

## Supporting information

Fig S1

Fig S2

Fig S3

Fig S4

Fig S5

Fig S6

Fig S7

Table S1

Table S2

Table S3

## Data Availability

The microarray data of human tissue from GSE2223, GSE4058, GSE4290, GSE13276, GSE68848 and GSE70231 were downloaded from the Gene Expression Omnibus (GEO) database. TCGA‐GBM database.
