# Supplementary figures and images for: Gene regulation network analysis reveals core genes associated with survival in glioblastoma multiforme
Source: J Cell Mol Med. 2020 Jul 21;24(17):10075–87. doi: 10.1111/jcmm.15615 (PMC7520335; doi:10.1111/jcmm.15615)

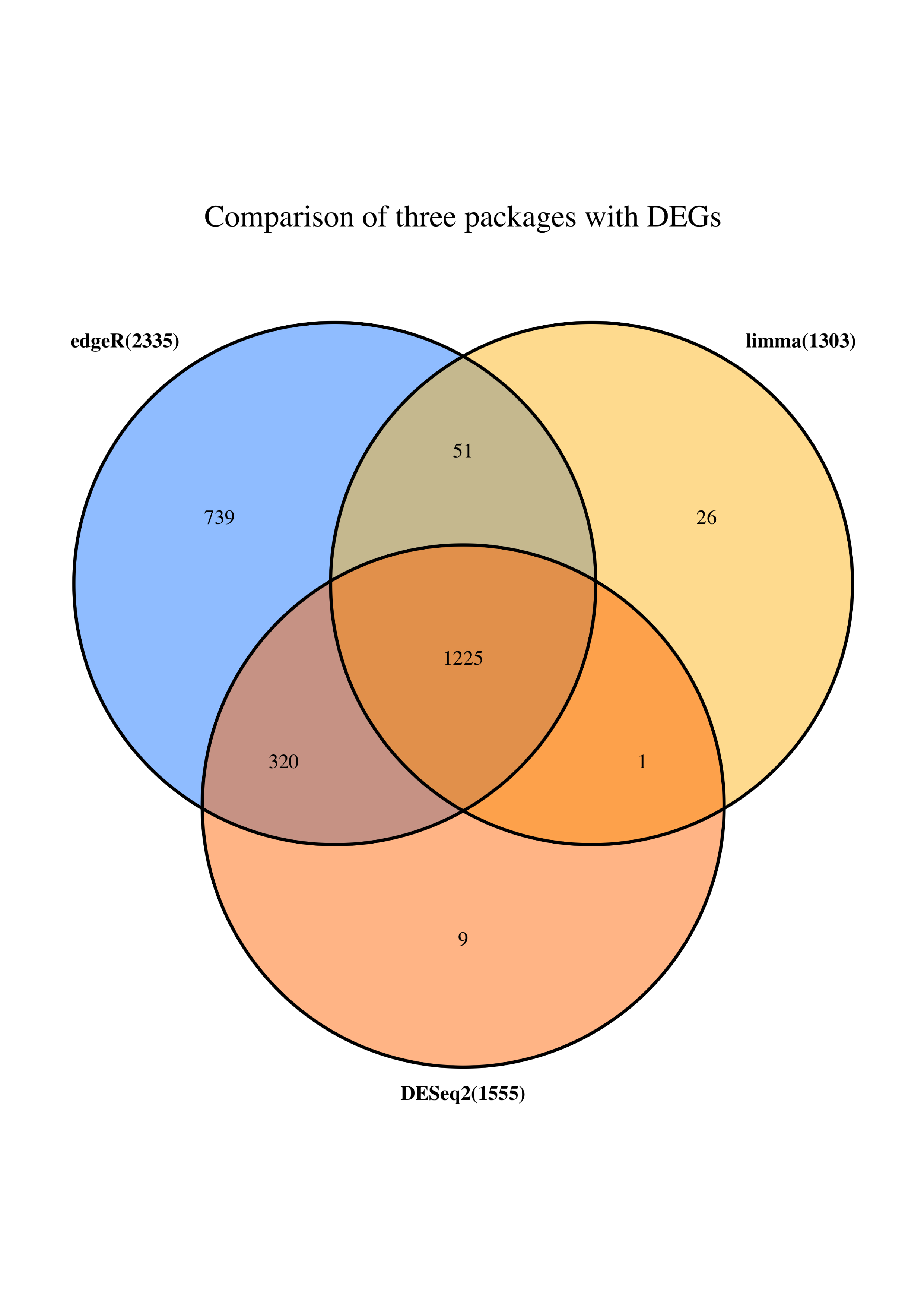

Supplement: Supplementary file 1 — Fig S1 [file JCMM-24-10075-s001.tif]

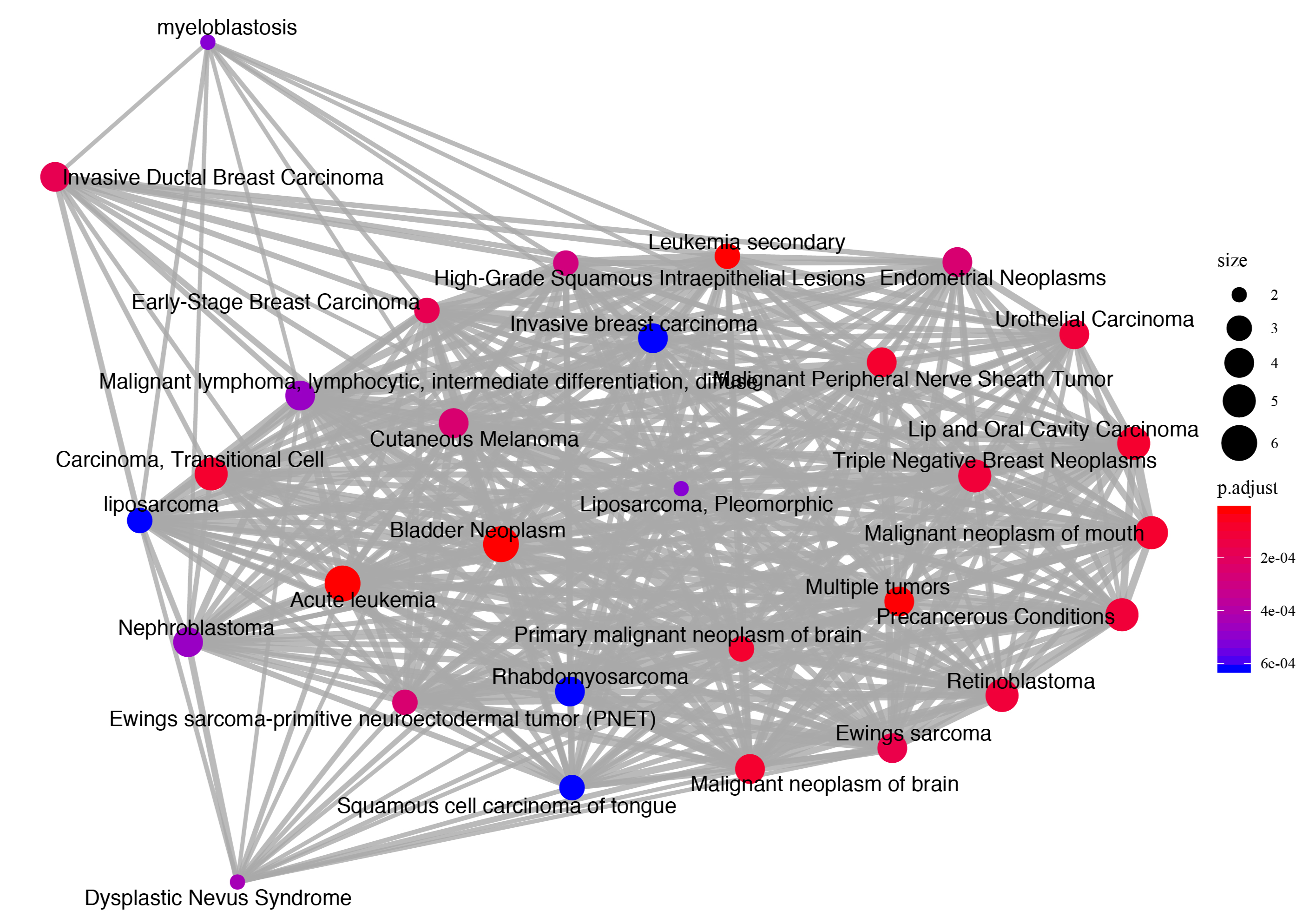

Supplement: Supplementary file 2 — Fig S2 [file JCMM-24-10075-s002.tif]

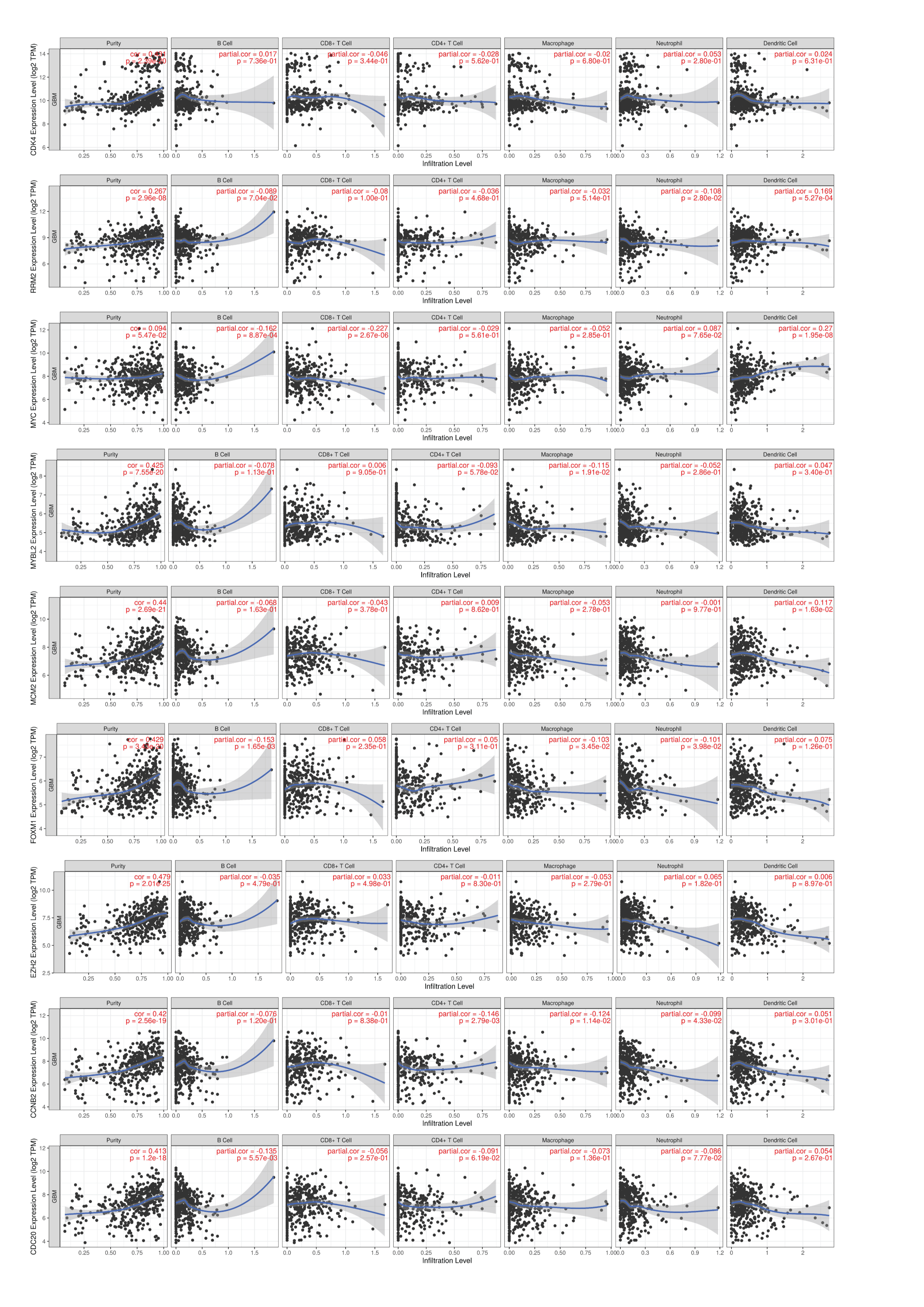

Supplement: Supplementary file 3 — Fig S3 [file JCMM-24-10075-s003.tif]

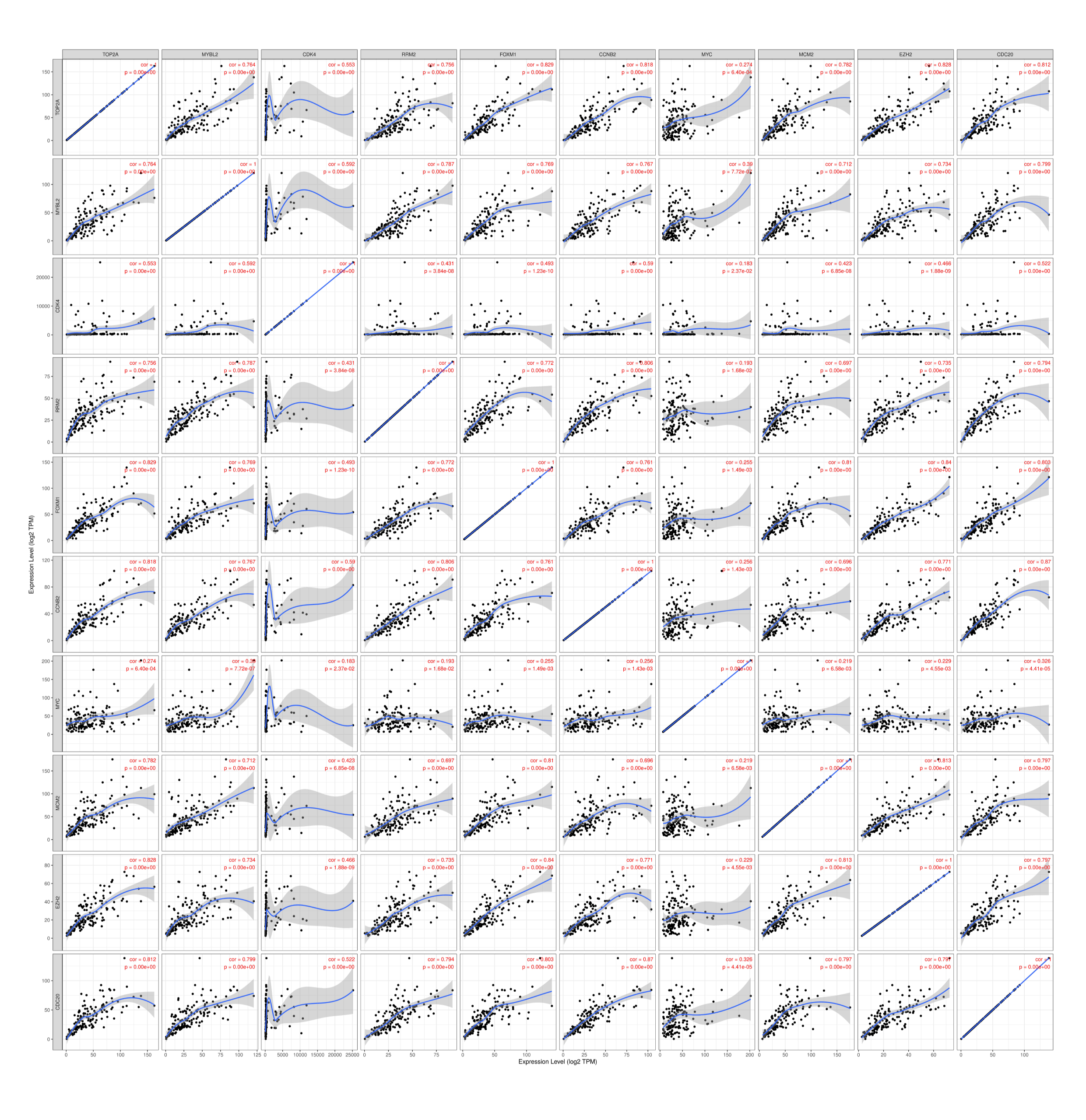

Supplement: Supplementary file 4 — Fig S4 [file JCMM-24-10075-s004.tif]

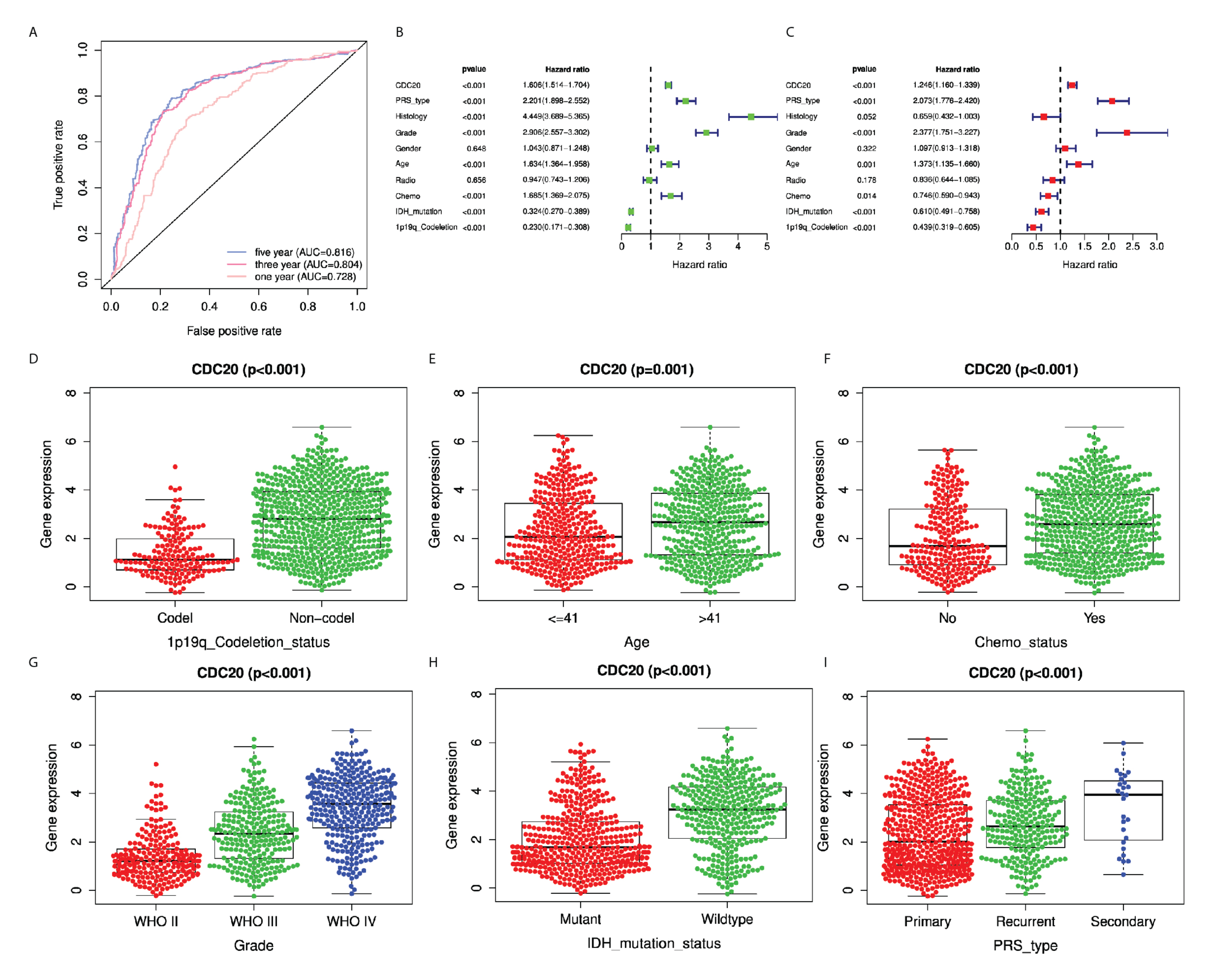

Supplement: Supplementary file 5 — Fig S5 [file JCMM-24-10075-s005.tif]

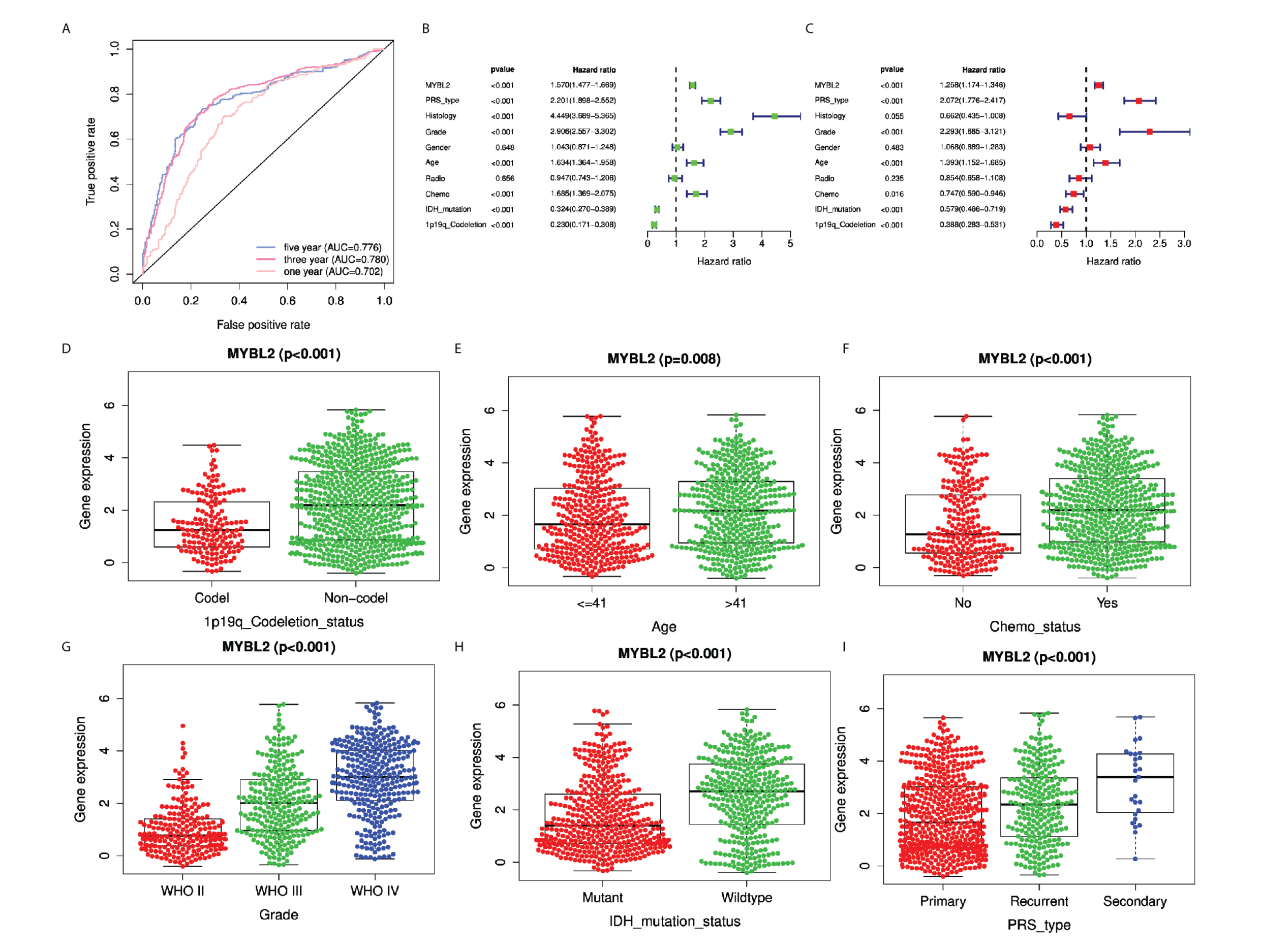

Supplement: Supplementary file 6 — Fig S6 [file JCMM-24-10075-s006.tif]

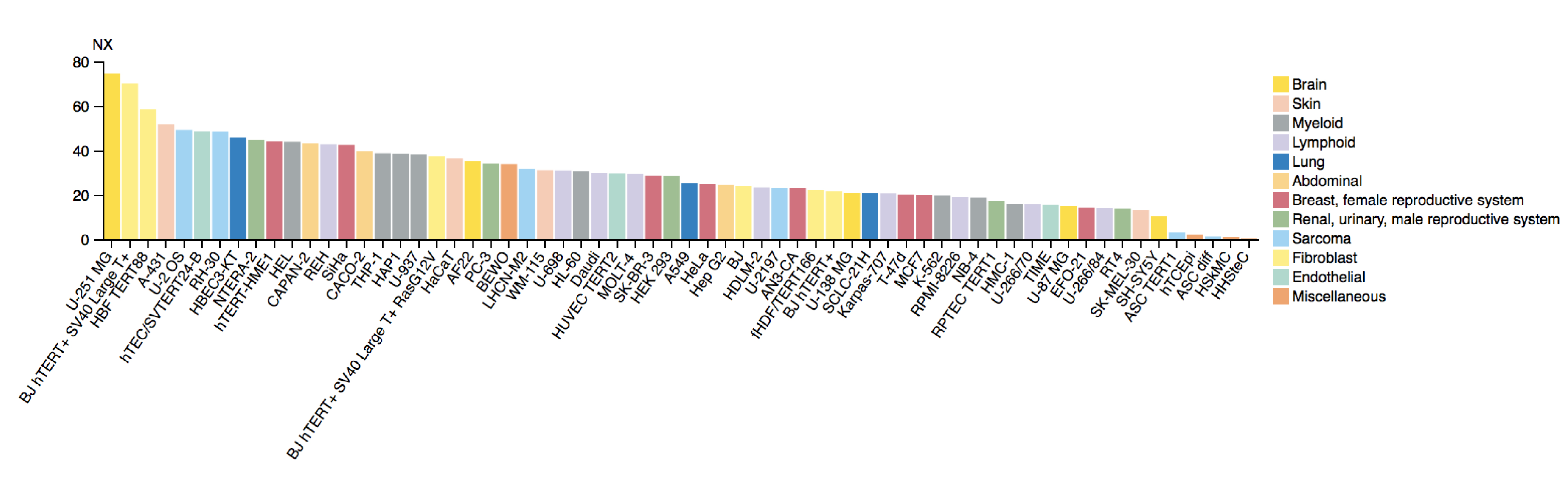

Supplement: Supplementary file 7 — Fig S7 [file JCMM-24-10075-s007.tif]
